# Using quality scores and longer reads improves accuracy of Solexa read mapping

**DOI:** 10.1186/1471-2105-9-128

**Published:** 2008-02-28

**Authors:** Andrew D Smith, Zhenyu Xuan, Michael Q Zhang

**Affiliations:** 1 Cold Spring Harbor Laboratory, Cold Spring Harbor, NY 11274, USA

## Abstract

**Background:**

Second-generation sequencing has the potential to revolutionize genomics and impact all areas of biomedical science. New technologies will make re-sequencing widely available for such applications as identifying genome variations or interrogating the oligonucleotide content of a large sample (*e.g*. ChIP-sequencing). The increase in speed, sensitivity and availability of sequencing technology brings demand for advances in computational technology to perform associated analysis tasks. The Solexa/Illumina 1G sequencer can produce tens of millions of reads, ranging in length from ~25–50 nt, in a single experiment. Accurately mapping the reads back to a reference genome is a critical task in almost all applications. Two sources of information that are often ignored when mapping reads from the Solexa technology are the 3' ends of longer reads, which contain a much higher frequency of sequencing errors, and the base-call quality scores.

**Results:**

To investigate whether these sources of information can be used to improve accuracy when mapping reads, we developed the RMAP tool, which can map reads having a wide range of lengths and allows base-call quality scores to determine which positions in each read are more important when mapping. We applied RMAP to analyze data re-sequenced from two human BAC regions for varying read lengths, and varying criteria for use of quality scores. RMAP is freely available for downloading at .

**Conclusion:**

Our results indicate that significant gains in Solexa read mapping performance can be achieved by considering the information in 3' ends of longer reads, and appropriately using the base-call quality scores. The RMAP tool we have developed will enable researchers to effectively exploit this information in targeted re-sequencing projects.

## Background

The main technological advances that accompanied the genomic and post-genomic eras are high-throughput sequencing and hybridization microarrays. Sequencing technology enabled scientists to obtain the full genomic sequence for many species, including the human and many model organisms. Sequencing technology is also being used to selectively re-sequence the human genome to detect genome variations such as single nucleotide polymorphisms or large-scale structural variations. Because understanding these variations can immediately impact medical sciences, making sequencing more efficient and accessible is imperative. However, traditional methodologies used to sequence the first mammalian genomes remain expensive, time consuming, and labor intensive.

Oligonucleotide microarray technology, used to interrogate the RNA or DNA content of a sample, has emerged as a widely accessible and effective tool for studying gene expression or detecting protein-DNA interactions (*i.e*. ChIP-chip). Microarrays have also been used to detect genome variations, like SNPs or structural variations. Array-based hybridization also has limitations. For example, probes can behave in highly non-uniform ways, and the effects of cross-hybridization and resolution limits are poorly understood. Although significant research efforts are focused on these problems, they remain inherent in all hybridization-based methods.

The new sequencing technology referred to as "second-generation" shows promise to eliminate many of the problems associated with traditional sequencing technology and also those with oligonucleotide microarray technology. Second-generation sequencers are able to sequence more quickly and at lower cost in terms of both money and labor. New sequencing technologies are developed to sequence at greater depth, meaning that a clone can be sequenced from a sample even when that clone exists at very low abundance (*i.e*. 1 molecule per cell).

Several second-generation technologies have been developed using diverse methods. Two recent second-generation sequencers are from 454 Life Sciences (Roche Diagnostics) and Solexa (Illumina). The 454 sequencers use an emulsion method for DNA amplification and a pyrosequencing protocol for sequencing by synthesis (SBS) at picolitre-scale volumes. Current 454 sequencers can produce 25–50 M nt of sequence in a single run, in the form of reads with length up to 500 nt (a number expected to increase), enabling this technology to be used for *de-novo *sequencing in addition to re-sequencing [1].

Solexa/Illumina 1G sequencers also use sequencing by synthesis, with DNA amplified on the surface of a flow cell, resulting in a random array of dense clusters [2]. The Solexa technology is faster and cheaper than that used in 454 sequencers, producing 1G nucleotides of sequence in one run but producing much shorter reads. Each individual read is roughly 25 to 50 bases in length (also expected to increase slightly in coming years). The Solexa sequencing technology has recently started producing breakthrough results. Research described in [3] and [4] employed Solexa sequencers to obtain high-resolution genomic maps of several histone modifications, as well as localization data for the DNA-binding proteins. Effectiveness of ChIP-sequencing has also been demonstrated by [5], who used Solexa sequencing to obtain locations of STAT1 binding sites in HeLa S3 cells before and following IFN-γ stimulation.

Mapping reads from the Solexa sequencer presents an obvious algorithmic challenge: tens of millions of reads must be mapped to a large (*e.g*. mammalian) genome in a reasonable amount of time. Strong efforts to design short-read mapping algorithms have resulted in methods that are effective in particular contexts. The mapping algorithm implemented as part of the Solexa analysis pipeline is named ELAND (Efficient Large-Scale Alignment of Nucleotide Databases). ELAND is optimized to map very short reads, with length at most 32 nt, and ignores the additional bases when the sequenced reads are longer. ELAND also only allows at most two mismatches between the read and the genomic sequence to which it maps, which will clearly be too few for longer reads. Despite these restrictions, ELAND remains very useful for many mapping tasks because it is extremely efficient. The SXOligoSearch algorithm (by Synamatix) can quickly map reads of varying length, using different criteria, with performance depending on both the read length and mapping criteria. The performance of SXOligoSearch depends on use of the proprietary SynaBASE data structure. This data structure is a heavily compressed and annotated index for the reference genome that retains all non-redundant information. The gains in mapping speed by using such a data structure come at a cost in terms of the memory required for the SynaBASE data structure. Extreme memory requirements of the data structure makes SXOligoSearch unsuitable for use on hardware available in most labs.

In most re-sequencing applications accuracy of mapping is a primary concern. We must know with great accuracy what part of the genome was actually sequenced. There are several reasons why it might be difficult to determine the location in the reference genome from which a read was derived, or even if a read was derived from the reference genome. These include problems with the experiments, such as sequencing errors or sample contamination. Mapping is also made more difficult by repeats in the genome, and by polymorphisms. While mapping algorithms cannot be expected to be robust to all such problems, effort should be made to make the algorithms as robust as possible.

Two sources of information with the potential to improve mapping accuracy are the 3' ends of longer reads, which are often ignored because they contain a higher frequency of errors, and the base-call quality scores. The quality scores describe the confidence of bases in each read. Sequencing quality scores, introduced in the Phred algorithm [6,7], assign a probability to the four possible nucleotides for each sequenced base. The Solexa analysis pipeline, for example, includes a program called BUSTARD to calculate quality scores. Because the bases with lower quality scores are more likely to be sequencing errors, any potential mapping for a read should be penalized less for mismatching at positions with lower scores. The quality scores are especially important for mapping longer reads, since the 3' ends of longer reads are known to have a higher frequency of sequencing errors.

To investigate whether these sources of information can be used to improve accuracy when mapping reads, we developed the RMAP tool, which can map reads having a wide range of lengths and allows base-call quality scores to determine which positions in each read are more important when mapping. The only requirement on the base-call quality scores for use in RMAP is that they increase monotonically with the inverse of the error probability for a particular base call. Our results indicate that significant gains in mapping performance can be achieved by considering the information in 3' ends of longer reads, and appropriately using the quality scores.

## Results and Discussion

### The mapping criteria

We designed RMAP to use two different mapping criteria, both based on approximate matching of the read and the reference genome. The first criterion is a simple count of mismatches between a read and the aligned genomic segment. Under this criterion, any unknown nucleotides in the reference genome (*i.e*. Ns) will induce a mismatch with any nucleotide. Uncalled positions, where the sequencing was unable to determine the nucleotide, also induce a mismatch. For a fixed read length, by allowing a greater number of mismatches, more reads can be mapped to reference genome. We refer to this simple mismatch criterion as RMAPM (RMAP using *mismatch *scores).

The second criterion, also based on mismatch-counts, makes use of the base-call quality scores. A cutoff for the base-call quality score is used to designate positions as either high-quality (HQ) or low-quality (LQ), depending on whether the quality score of the highest-scoring base at that position exceeds the cutoff. Low-quality positions always induce a match (*i.e*. act as wild-cards). To prevent the possibility of trivial matches, a quality control step eliminates reads with too many low-quality positions. As with the first criterion, mapping accuracy can be controlled by manipulating the number of allowed mismatches when mapping. But for the second criterion, manipulating the quality-score cutoff provides another means of adjusting sensitivity and specificity, and allows positions to contribute when they are of high-quality, but not be penalized if they are low-quality. We refer to this criterion as RMAPQ (RMAP using *quality *scores).

### Evaluating mapping accuracy

#### Measuring mapping accuracy

In measuring mapping accuracy, we want to quantify both sensitivity and specificity by using reads sequenced from DNA samples from selected genomic regions instead of the entire genome. By mapping those reads to the genome, we can evaluate how accurately they are mapped to the target region. However, there are theoretical and practical limits to how well these can be measured. Inability to map a read correctly can be attributed to sequencing errors (arising from any part of the experiment), to variation between the sampled genome and the reference genome, or can result from ambiguities caused by repeats in the reference genome. These diverse sources of error make it difficult to measure accuracy in terms of traditional sensitivity and specificity.

Ambiguous reads, under a given mapping criterion, are reads that map to more than one location in the reference genome. Reads that map to a single location are called uniquely mappable (or simply mappable) reads. All reads that are not mapped uniquely to some location in the reference genome are said to be unmappable (which includes ambiguous reads). We define target region coverage (or simply coverage) as the number of bases in the target region covered by at least one mappable read, divided by the total number of bases in the target region. In order to compare the coverage values for different read lengths, we use only the first base of each read to represent that read. By counting bases covered, rather than number of reads that map to the target region, greater target region coverage is achieved when the reads map uniformly in the target region. We define mapping *selectivity *as the number of mappable reads that map inside the target region, divided by the total number of mappable reads. A read is said to map inside the target region if any part of the read overlaps the target region. The selectivity shows how well the mapping criterion places mappable reads inside the target region. In this study, when we refer to mapping accuracy, we are referring to both coverage and mapping selectivity (and we formally treat accuracy as the mean of these two measures).

#### Evaluation data

We used the data from samples of two BACs provided for sequencer validation by Solexa, which covers 162 kb of the chromosome 6 MHC region in an A1-B8-DR3 alternate haplotype assembly based on sequence data from the COX library [8]. We chose one lane of reads sequenced by the 1G sequencers at the CSHL Genome Center. The total number of raw 36 nt reads in this data set is 3.4 million, with the quality score of each base ranging from -5 to 40 (as called by the BUSTARD program from the Solexa analysis pipeline). As reference genome we used hg18, all chromosomes except chr6, which we replaced entirely with chr6_cox_hap1, the A1-B8-DR3 alternate haplotype assembly. We chose to include this alternate haplotype in the reference because it is the origin of the BAC that was sequenced. We excluded the ordinary chr6 because it has high similarity with chr6_cox_hap1, and including both of these would have resulted in a high proportion of reads mapping ambiguously to chr6 and chr6_cox_hap1 (see additional file 1).

#### Evaluation procedure

To investigate how information is distributed within the reads, we ran RMAP on all reads with lengths ranging from 25–36 nt, allowing mismatches in the range of 0 to 10, and using both the RMAPM and RMAPQ criteria. For the RMAPQ criterion, we chose {4, 8, 12, 16, 20, 24} as the set of quality-score cutoffs to evaluate. Reads with fewer than 10 contiguous HQ bases (*i.e*. bases scoring above the quality-score cutoff) were considered unmappable and removed from consideration, as the algorithm requires a minimum number of high-quality bases for efficiency (see Methods section for details). Any read lacking 10 consecutive high-quality bases would likely have a very high overall amount of error.

### Mapping longer reads with more mismatches increases accuracy

**Figure 1:**
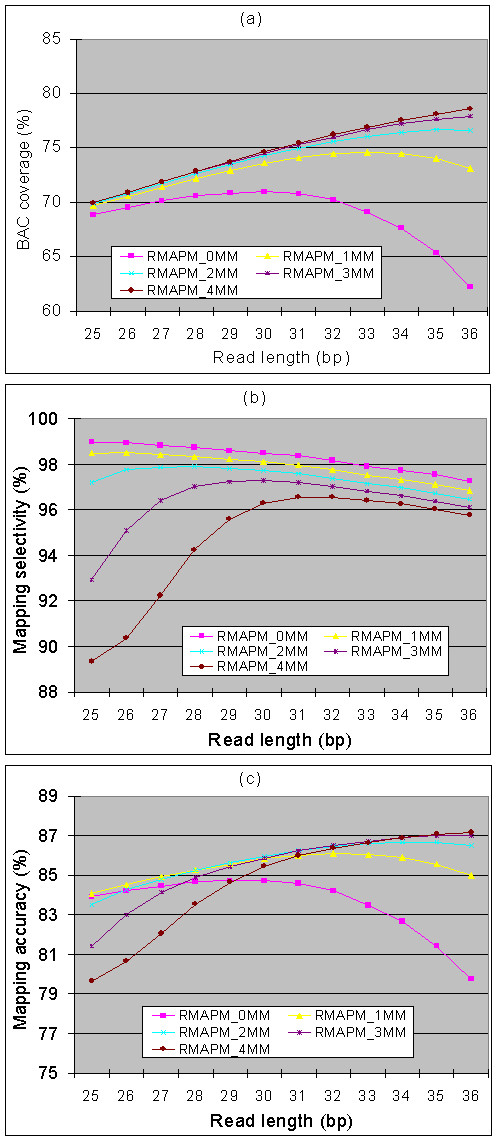
**Comparison of mapping accuracy of RMAPM criterion under different parameter combinations**. Comparison of mapping accuracy for reads of different lengths, and allowing different numbers of mismatches without using quality scores. Both the target (BAC) region coverage (a) and the mapping selectivity (b) are displayed. The mean of these two measures is presented in (c) as mapping accuracy. Standard error of displayed values was always ≤ 1.0% and usually < 0.1%, as estimated by mapping reads obtained from the second lane of the same sequencing run of the same BAC regions (this applies also to values in Figure 2).

The Solexa sequencer can produce reads of more than 50 bases, and longer reads contain more sequence information. Although it is known that the quality of sequenced bases in reads decreases toward the 3' end of the read, especially as read length increases, it remains to be shown how much useful information may still exist in bases at the 3' ends of longer reads. Making use of any additional bases is only expected to improve mapping accuracy if the additional bases contain information of sufficient quality. When the BAC reads were mapped to the human genome using the RMAPM criterion, with length from 25–36 nt, and different number of allowed mismatches, we found that there is generally a great deal of information in 3' end bases up to 36 nt. These results are presented in Figure 1 and Supplementary Table 1 (see additional file 2) (We remark that the accuracy of RMAP using the RMAPM criterion for reads shorter than 32 nt, allowing at most 2 mismatches, is the same as that of ELAND). The BAC coverage always increased with length of mapped reads, except when only one or zero mismatches are allowed. The mapping selectivity decreases monotonically with read length when zero or at most one mismatch is allowed. When multiple mismatches are allowed, the mapping selectivity first increases with read length, then decreases slightly. Taking the mean of these two measures as overall mapping accuracy, we see that the combined target region coverage and mapping selectivity is maximized when read length is 36 nt and up to 4 mismatches are allowed. Comparing read lengths between 25 nt and 36 nt, the mapping selectivity increases 6.4% while the BAC region coverage increases 8.6%. By extrapolating our results, even greater improvements are expected as read lengths increase beyond 36 nt.

### Using quality score information increases accuracy

**Figure 2:**
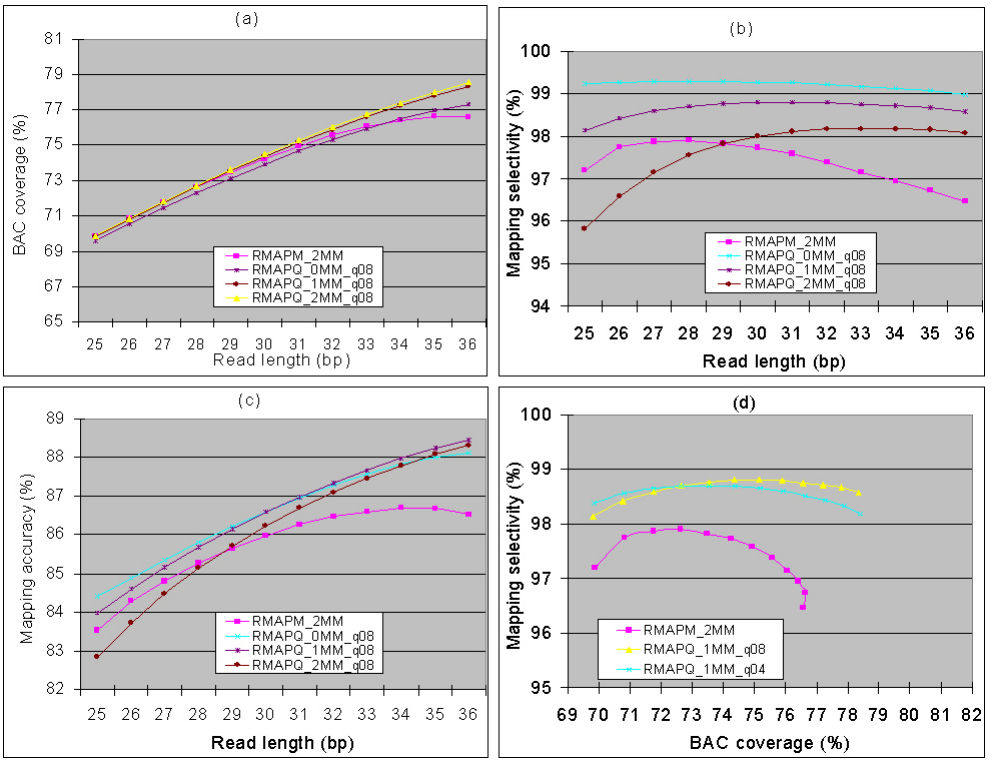
**Mapping accuracy of RMAPQ criterion under varying parameters**. Reads with length from 25–36 nt were mapped and 0,1, or 2 mismatches were allowed at high quality bases defined by quality score cutoffs of 4 (d) or 8 (a-d). For reference, mapping performance of RMAPM criterion with at most 2 mismatches is also shown. (a) The BAC coverage; (b) the mapping selectivity; (c) the overall mapping accuracy (equal to the mean of the BAC coverage and selectivity).(d) 2-D performance comparison in both BAC coverage and selectivity of RMAPM and RMAPQ.

We tested different cutoffs for defining high quality bases to estimate the ability of the RMAPQ criterion to most effectively use the quality information in the reads. We achieve better mapping performance in both BAC coverage and mapping selectivity when using longer reads with high quality score cutoff of 4 to 24 than without using quality score filtering (See Figure 2, and additional file 2). This is due to a larger number of reads being mapped unambiguously to the genome by the RMAPQ criterion, and a larger number of those being mapped to the target (BAC) regions. The best mapping accuracy was achieved when reads were of length 36 nt, a quality-score cutoff of 8 was used, and when at most one mismatch was allowed. For these settings, the mapping selectivity further improved almost 3% with the same BAC coverage, compared with the best accuracy of RMAP using the RMAPM criterion. These results demonstrate that the way in which the quality scores are incorporated into RMAP results in improved accuracy.

The reads derived from the BACs provided by Solexa for the purpose of validation are of very high quality. For a given 162 Kb BAC region, most of these 3.4 million reads can be mapped almost perfectly. This introduces a saturation effect, leaving limited space for improvement, as highly accurate mapping is achieved easily. RMAP still makes significant improvements within this narrow range. In many applications, without this ceiling effect, the improvement is expected to be even more pronounced.

We also compared the mapping accuracy of RMAP with another available, but presently unpublished, method called MAQ [9]. Using default parameter setting, we ran MAQ to map the set of 3.4 M reads from the BAC. Although MAQ had speed and memory usage similar to RMAP, we found MAQ to have lower mapping accuracy (see additional file 3) when allowing at most 2 mismatches.

## Discussion

Widely-accessible second-generation sequencing technologies promise to revolutionize many areas of bio-medical research. In addition to *de novo *sequencing of new species, these technologies make targeted re-sequencing a reality. Re-sequencing will provide more accurate means of interrogating the oligo-nucleotide content of samples, and of identifying important genome variations, such as disease-related SNPs. Mapping reads to genomes is a critical step in re-sequencing data analysis, and both the algorithmic and software technology must keep pace with surging advances in the throughput of sequencing instruments.

In order to maximize the use of available information in mapping Solexa reads, we developed the RMAP tool, which incorporates base-call quality scores to improve accuracy. RMAP responds to an urgent need for such an algorithm by providing both the accuracy to handle emerging mapping tasks. Our results in applying RMAP have shown that more reads can be mapped into the target regions when using the RMAPM criterion to map longer reads and allow more mismatches. We have also shown that the way in which quality scores are used in RMAP (the RMAPQ criterion) significantly increases both coverage and mapping selectivity.

Although second-generation sequencing technology is currently producing many important results, there is still little understanding of how this technology should behave with respect to sequencing errors and what are the general properties of typical re-sequencing data sets. As more knowledge accumulates about typical results from this new sequencing technology, more information can be incorporated into algorithms for mapping reads and other associated analysis tasks.

In theory we could move toward an ideal mapping criterion by predictive modeling, where a model would be trained to identify the location from which each read was derived. Although the best mapping criteria may not be amenable to high-throughput computation, some approximation of those criteria could be developed. Cross-validation and the use of a wide range of data sets could be used to ensure that the trained criteria are sufficiently general. In practice such a procedure would require high-quality training data, and an extreme amount of computing time to train and evaluate such models.

RMAP does not consider insertions or deletions (indels), which are potentially important in certain sequencing applications (*e.g*. indel polymorphisms). The straight-forward strategy for handling indels is to extend initial seed matches using a Smith-Waterman-style alignment, as is commonly done in database search programs like BLAST [10]. For short reads, with length ≤ 50 bases, providing this greater flexibility will require careful investigation into scoring the alignments, because using simple scoring schemes for indels may result in higher rate of false-positive mappings and ambiguities. In addition, because indels have nothing analogous to the base-call quality scores, it will be more difficult to distinguish errors from real genotypic variations during the mapping stage of analysis. We have observed that data sets containing a high proportion of low-complexity reads, such as single nucleotide repeats, cause a decrease in execution speed of RMAP. This is explained by such low complexity seeds resulting in a large increase in the number of full comparisons (between reads and regions of the reference genome), that are induced by these highly-common seeds. The exclusion strategy used in RMAP is also heavily used by popular programs for searching sequence databases. Research effort toward improving the structure of seeds used in these algorithms has also led to improvements in the algorithms themselves [11], and similar improvements might be observed by developing seeds specifically for the short read mapping problem.

The filtration strategy used in RMAP can also be extended to "multiple filtration", as described by [12]. This uses multiple criteria for excluding possible mappings, and would result in the algorithm performing fewer full comparisons between reads and genomic regions. Unlike many other applications of approximate matching, the extreme volume of data that must be mapped means that algorithms for mapping reads must be conscious of the memory required. In the framework of RMAP, the most straight-forward implementation of multiple filtration would use larger (multidimensional) or additional hash-tables, which generally requires a great deal more memory. For short reads, as produced by the Solexa sequencer, the full comparisons can be computed very fast, so another concern is keeping the work required to implement more powerful filtration less than that required to do the extra comparisons.

Aside from further processing on the set of reads, efficiency improvements can be gained by indexing the reference genome. This is the strategy used by SXOligoSearch, through the proprietary SynaBASE data structure. Because the reference genome will likely remain static for many mapping tasks (*e.g *several experiments using the hg18 genome), this indexing can be done off-line, so the time required to build such an index is not important. The usual problem with indexing the genome is that such index structures are often several times larger than the genome itself. The more information built into the index to facilitate searches, the larger the index must be. As stated above, hardware currently available for mapping usually has a few GB of memory, and this restricts the kinds of indexing that can be done on entire genomes. To make genome index structures practical, they must either be small, or support on-line processing so that only a small portion of the structure must reside in memory at once. It is likely that special purpose data structures for indexing the genome can be developed to fit these requirements, and greatly improve efficiency of mapping reads. Specifically with respect to RMAP, adding any means of eliminating exact genomic repeats during mapping will greatly improve efficiency.

## Conclusion

Our results indicate that significant gains in Solexa read mapping performance can be achieved by considering the information in 3' ends of longer reads, and appropriately using the base-call quality scores. The RMAP tool we have developed will enable researchers to effiectively exploit this information in targeted re-sequencing projects.

## Availability and Requirements

The RMAP tool is freely available for downloading at .

## Methods

### Design of the RMAP algorithm

In this section we describe the algorithmic strategy used in RMAP. We treat the mapping problem as approximately matching a set of patterns in a text – the set of patterns being the reads, and the text being the genome. This problem has been well studied, and several general algorithmic strategies have emerged for solving it (see [13] for a detailed treatment). The major motivation for developing the RMAP algorithm was to incorporate base-call quality scores to weight mismatches and improve mapping accuracy. In addition to having high mapping accuracy, RMAP was designed under the restrictions that it must be capable of (1) mapping reads with length exceeding 50 bases (for the applications discussed in the introduction), (2) allowing the number of mismatches to be controlled (not being restricted to a small fixed number), and (3) completing mapping tasks under reasonable time constraints on widely available computing hardware.

The algorithm implemented in RMAP uses the filtration method described by [14]. For reads of length *n*, and mapping with up to *k *mismatches, each read is partitioned into *k *+ 1 contiguous *seeds *(each seed is a substring of the read, and has length ⌊*n*/(*k *+ 1)⌋). Because there can only be *k *mismatches in a mapping, and there are *k *+ 1 seeds for each read, any mapping must have at least one seed with no mismatches. The algorithm first identifies locations in the genome where the seeds match exactly. Exact matching can be done much more quickly than approximate matching, and evaluating the approximate match between a read and a genomic region only needs to be done for those regions surrounding an exactly-matching seed.

To efficiently implement the filtration strategy, RMAP pre-processes the set of reads, building a hash-table (which we refer to as the seed-table) indexed by the seeds. The table entry for a particular seed lists all reads containing that seed, along with the offset of that seed within the read. For a set of *r *reads, each having length *n*, if *k *mismatches are allowed in the search, the seed table has size *O*(4^*n*/*k *^+ *rk*). The mapping proceeds by scanning the genome, with a window of size equal to the seed size. Each segment of the genome is tested as a seed by hashing that segment to determine the set of reads that must be compared in their entirety with a larger genomic region surrounding the segment of the genome currently being scanned. This is a common strategy to implement the filtration stage of approximate matching. The influence of the size of the genome in the time complexity of RMAP is therefore linear, and importantly the space complexity of RMAP is independent of the size of the genome.

The step of comparing the full read to portions of the genome where a seed has been found is implemented to require time that is logarithmic in the length of the reads. The comparison takes advantage of bit-wise operations, and the reads are encoded in a binary format (see additional file 4 for supplementary method). A series of logical operations produce a vector indicating the locations of mismatches between the read and the genomic segment being compared, and the weight of the bit-vector indicating mismatches computed using a well-known technique described by [15].

RMAP is sufficiently fast that several million reads can be mapped to a mammalian genome in one day on a computer with a single processor. No portion of the reference genome is maintained by RMAP, and the size of the seed table dominates the space requirements. Because these requirements are sufficiently small, RMAP can be run on widely available hardware. This includes the nodes typically used in cluster computers, and allows the processing to be easily and effectively parallelized by simply partitioning the set of reads. On a test data set generated by randomly sampling one million 50 nt segments (simulated reads) from the hg18 genome, and randomly changing up to 4 bases in each read, our current implementation of RMAP was able to map the reads back to the hg18 genome in 140 minutes using roughly 620 MB of memory.

## Authors' contributions

ADS, XZ and MQZ conceived the research; ADS and XZ conducted the research and wrote the article. All authors have read and approved the final manuscript.

## Supplementary Material

Additional file 1 Mapping performance comparison by using either chr6 or its COX alterantive haplotype. Comparing the mapping performance for RMAPM and RMAPQ when using either chr6 or its COX alterantive haplotype assembly. Click here for file

Additional file 2 Reads mapped in genome and BACs by using different mapping criteria. Number of reads mapped to genome and BACs by using RMAPM or RMAPQ with different read length and maximum number of allowed mismatches. Click here for file

Additional file 3 Mapping performance comparison with MAQ. Comparing the mapping performance between RMAPM, RMAPQ with MAQ by using different read length. Click here for file

Additional file 4 Counting mismatches between reads and genomic sequence. The method used for counting mismatches between reads and genomic sequence. Click here for file
