# Supplementary material for: Using quality scores and longer reads improves accuracy of Solexa read mapping
Source: BMC Bioinformatics. 2008 Feb 28;9:128. doi: 10.1186/1471-2105-9-128 (PMC2335322; doi:10.1186/1471-2105-9-128)
Supplement: Additional file 3 — Mapping performance comparison with MAQ. Comparing the mapping performance between RMAPM, RMAPQ with MAQ by using different read length. [file 1471-2105-9-128-S3.pdf]

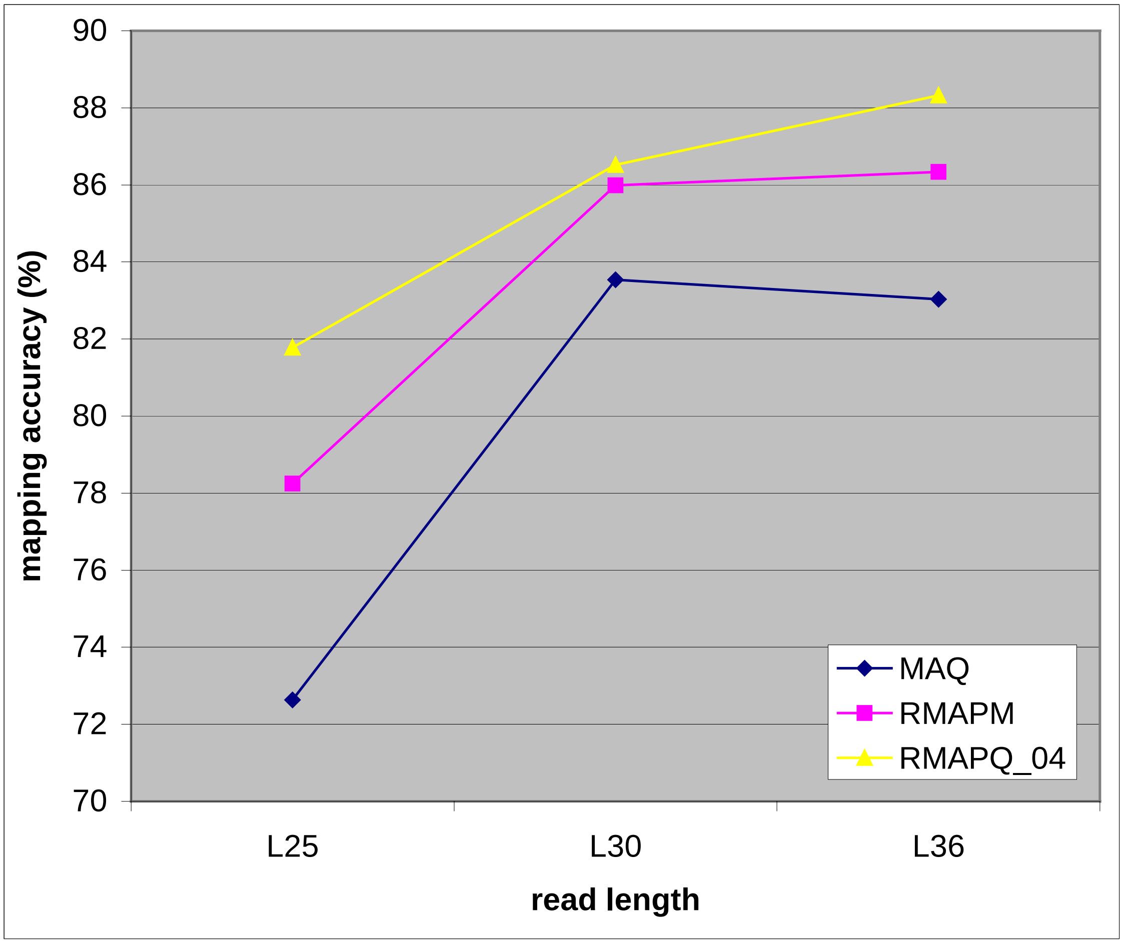

Supplementary figure 1: Accuracy comparison with MAQ: mapping accuracies of MAQ, RMAPM and RMAPQ. Three different read lengths were used {25bp, 30bp, 36bp}. Default parameter setting is used for MAQ. Two mismatches are allowed for RMAPM and RMAPQ, and quality score cutoff for RMAPQ is 4. Mapping accuracy = (coverage + selectivity)/2 (see main text).
