# Supplementary material for: Using quality scores and longer reads improves accuracy of Solexa read mapping
Source: BMC Bioinformatics. 2008 Feb 28;9:128. doi: 10.1186/1471-2105-9-128 (PMC2335322; doi:10.1186/1471-2105-9-128)
Supplement: Additional file 4 — Counting mismatches between reads and genomic sequence. The method used for counting mismatches between reads and genomic sequence. [file 1471-2105-9-128-S4.pdf]

Supplementary Information for: Using quality scores and longer reads improves accuracy of Solexa read mapping

Andrew D Smith Zhenyu Xuan Michael Q Zhang

Supplementary Methods

Counting mismatches between reads and genomic sequence. In the RMAP program the basic comparison between a read and any genomic region involves counting the number of mismatches between those two strings. Since all of the reads, as well as the genome, are stored in a binary format, a few machine operations (mainly involving XORs) can produce a machine word that is essentially a bit vector having a ’1’ at every position where the two strings differ.

To count the number of ’1’s in such a bit vector, we use a technique that employs bit parallelism. Here is some C code for the function:

```
unsigned int count_ones(unsigned int bits) {
  // ‘‘bits’’ is a machine word with a ’1’ where the read differs from the
  // genomic region it is being compared with
  bits = ((bits & 0xAAAAAAAAAAAAAAAA) >> 1)  + (bits & 0x5555555555555555);
  bits = ((bits & 0xCCCCCCCCCCCCCCCC) >> 2)  + (bits & 0x3333333333333333);
  bits = ((bits & 0xF0F0F0F0F0F0F0F0) >> 4)  + (bits & 0x0F0F0F0F0F0F0F0F);
  bits = ((bits & 0xFF00FF00FF00FF00) >> 8)  + (bits & 0x00FF00FF00FF00FF);
  bits = ((bits & 0xFFFF0000FFFF0000) >> 16) + (bits & 0x0000FFFF0000FFFF);
  return ((bits & 0xFFFFFFFF00000000) >> 32) + (bits & 0x00000000FFFFFFFF);
}
```

In case this doesn’t make sense to you, the constants like “0xAAAAAAAAAAAAAAAA” are 64-bit hexadecimal numbers, the “¿¿” operator is a binary right-shift operator, and “&” is the bit-wise AND operator. Note that this code would only work on a 64-bit machine, because the mask constants (they start with “0x”) are too large to fit in a 32-bit word. The technique counts ’1’s by adding up the number of ’1’s in parallel for different segments of the bit vector. The first line:

```
  bits = ((bits & 0xAAAAAAAAAAAAAAAA) >> 1)  + (bits & 0x5555555555555555);
```

starts with the vector of ’1’s where there are mismatches, and produces a vector with the counts of ’1’s in adjacent pairs of positions. We illustrate an example that assumes an 8-bit word. The code for 8 bits would look like:

```
unsigned int count_ones(unsigned int bits) {
  bits = ((bits & 0xAA) >> 1)  + (bits & 0x55);
  bits = ((bits & 0xCC) >> 2)  + (bits & 0x33);
  return ((bits & 0xF0) >> 4)  + (bits & 0x0F);
}
```

Suppose we begin with the following:

```
11010010
```

Then the line would produce

$$((\texttt{11010010}\&\texttt{10101010}) >> \texttt{1}) \Rightarrow \texttt{01000001}$$

on the left side of the sum (0xAA is 10101010 in binary), and

$$(\texttt{11010010}\&\texttt{01010101}) \Rightarrow \texttt{01010000}$$

on the right side of the sum (Ox55 is 01010101 in binary). Taking the addition gives the following result, in binary:

$$\begin{array}{r} 01\ 00\ 00\ 01 \\ +\ 01\ 01\ 00\ 00 \\ \hline 10\ 01\ 00\ 01 \end{array}$$

Notice that because the part "(bits & 0xAA)" was shifted to the right, the summands have no '1' bits in the 1st, 3rd 5th or 7th bits (big endian). The result is that bits 1 and 2 store the number of '1's in the first two positions of the original bit vector; bits 3 and 4 store the number of '1's in the 3rd and 4th positions of the original bit vector, and similar for the other pairs of adjacent positions.

Now "`bits`" has the binary value 10010001, and we will go through the second line of the code for an 8-bit word. On the left of the sum we obtain

$$((\texttt{10010001}\&\texttt{11001100}) >> \texttt{2}) \Rightarrow \texttt{00100000}$$

(0xCC is 11001100 in binary); on the right of the sum we have

$$(\texttt{10010001}\&\texttt{00110011}) \Rightarrow \texttt{00010001}$$

(0x33 is 00110011 in binary). Then these two are added together, which looks like this:

$$\begin{array}{r} 0010\ 0000 \\ +\ 0001\ 0001 \\ \hline 0011\ 0001 \end{array}$$

The binary number represented in just the first four bits of the result (*i.e.* 0011) is exactly the number of '1's in the first four positions of the original bit vector. The binary number represented in the other four bits of the result (*i.e.* 0001) is exactly the number of '1's in the other four positions of the original bit vector.

Now we have a value of "00110001" in "bits", and we will do the final line of code:

```
return ((bits & 0xF0) >> 4)  + (bits & 0x0F);
```

The left side of the sum gives the following:

$$((\texttt{00110001}\&\texttt{11110000}) >> \texttt{4}) \Rightarrow \texttt{00000011}$$

(0xF0 is 1111000 in binary); on the right of the sum we get

$$(\texttt{00110001}\&\texttt{00001111}) \Rightarrow \texttt{00000001}$$

The binary addition of these two numbers looks like

$$\begin{array}{r} 00000011 \\ +\ 00000001 \\ \hline 00000100 \end{array}$$

and 00000100 is 4 in decimal. This value is returned from our 8-bit function when applied to 11010010, and it is exactly the number of ’1’s.

This technique is well known (computer science “folklore”), and can be found in *Hacker’s Delight* by Henry S Warren Jr. (Addison-Wesley, 2003).
